# Optimization and preparation of a compound cod liver oil film former agent: an orthogonal design

**DOI:** 10.3389/fphar.2024.1296448

**Published:** 2024-03-01

**Authors:** Yao He, Ying Zhang, Zheng Li

**Affiliations:** ^1^ Pharmacy Department, Chongqing Emergency Medical Center, Chongqing University Central Hospital, Chongqing, China; ^2^ Chongqing Key Laboratory of Emergency Medicine, Chongqing Emergency Medical Center, Chongqing University Central Hospital, Chongqing, China; ^3^ Pharmacy Department, The Fifth People’s Hospital of Chongqing, Chongqing, China

**Keywords:** orthogonal experimental design, recurrent aphthous stomatitis, polyvinyl alcohol, cod liver oil, dexamethasone acetate, metronidazole

## Abstract

**Background:** Cod liver oil has anti-inflammatory properties and could help regulate recurrent aphthous stomatitis (RAS). An orthogonal experiment was used to evaluate and improve the dosage form of compound cod liver oil, which has replaced the previously used liniment preparation based on film method.

**Methods:** An orthogonal experiment was adopted, and the appearance and film-forming time of the film coating agents were used as indicators. The optimal ratio in the preparation process for the compound cod liver oil film agent was then optimized. A method for determination of compound cod liver oil film was established using High-Performance Liquid Chromatography (HPLC).

**Results:** The results indicate that the blank films prepared using 55 mg polyvinyl alcohol (PVA) (PVA low), 45 mg of PVA (PVA medium), and 10 mg glycerol had the optimal performance, which was defined as PVAa. The drug-carrying film prepared from 3 mL PVAa (i.e., film-forming material with the optimal proportion), 30 mg dexamethasone acetate, and 30 mg metronidazole had the optimal performance. The verified sample has a complete and smooth appearance, uniform thickness and color, and no evident bubbles, which meets the requirements for a film agent defined in the Chinese Pharmacopoeia, 2020 edition. HPLC was used to determine the major components: dexamethasone acetate, metronidazole, and dyclonine hydrochloride, and the optimal separation effect was obtained. The method has advantages of good specificity, good linear results, high recovery rate, and good repeatability.

**Conclusion:** This study proposes an optimized compound cod liver oil film former agent and preparation method. The results indicate that the compound cod liver oil film former agent had good performance, reflecting the high feasibility of this research method. The detection method of compound cod liver oil film was established by HPLC. The method was feasible, and the validity and stability of the formulation and preparation technology were guaranteed. The role of the newly developed agent in patients with RAS should be investigated further.

## 1 Introduction

Recurrent aphthous stomatitis (RAS), also known as recurrent aphthous ulcers or canker sores, is one of the most prevalent ulcerative diseases of the oral mucosa (Giannetti et al., 2018; Rivera, 2019). RAS affects approximately 5%–25% of the global population and predominantly women from higher socioeconomic groups (Nalbantoglu and Nalbantoglu, 2020). It is characterized by multiple recurrent small round or ovoid ulcers with circumscribed margins, which may vary in size (Ghali and Abdulhamed, 2022). Aphthous ulcers are usually extremely painful for the first 4–5 days, during which they can interfere with eating and speaking. The exact etiology of RAS remains unclear; however, it is considered a multifactorial disease. Evidence has shown that emotional stress, allergies, local trauma, spicy food, and trace element or vitamin deficiencies are potential risk factors (Edgar et al., 2017; Rodriguez-Archilla and Raissouni, 2018; Mao et al., 2023).

Minor aphthous stomatitis can heal spontaneously within 4–14 days without scarring (Rivera et al., 2022), whereas major aphthous stomatitis, especially when the ulcers are >1 cm in diameter, results in long-lasting lesions that can scar (Krawiecka et al., 2017). Oral lesions can seriously interfere with the normal daily activities of patients, as they may cause severe difficulties with chewing, speaking, and swallowing (Chen, 2021). Numerous agents and methods are available for the treatment of RAS, and their objectives are primarily pain relief, promotion of wound healing, and reduction of frequency (Mao et al., 2022). However, most of the medicinal products on the market are western medicines, and few agents have a definite effect on RAS management and meet the treatment requirements of patients (Hamedi et al., 2016). The combined use of multiple agents has a considerable impact on patients’ medication compliance and increases treatment costs. This may also cause adverse reactions, recurrence, and other disadvantages, which makes clinical usage questionable. Moreover, physical therapy is inconvenient for patients, and the cost of treatment is high. Furthermore, the treatment effects do not differ significantly; therefore, it is often not the first choice for patients with RAU.

Cod liver oil is a rich source of omega-3 fatty acids with anti-inflammatory properties, and it could regulate various diseases, including RAS (Yang et al., 2019). The compound cod liver oil liniment mainly comprises cod liver oil, tetracycline powder, tetracaine, and dexamethasone (Li and Quan, 2005). The curative effect has a decreasing trend annually in clinical practice.

The aim of the present study was to optimize the dosage form of the compound cod liver oil, which improves liniment film preparation. The authors hypothesized that such modification could minimize washing away of active drug ingredients by saliva and poor local adhesion, and improve drug stability and bioavailability. An orthogonal experiment approach was adopted, and the appearance and film-forming time of the film coating agents were used as the indicators. Furthermore, the ratios in the preparation process for the compound cod liver oil film agent were optimized.

## 2 Materials and methods

### 2.1 Instruments and reagents

KQ-100B ultrasonic cleaner (Kunshan ultrasonic instruments Co., Ltd., Kunshan, China); AFA-IV automatic coating machine (Dongguan Dalai Instrument Co., Ltd., Dongguan, China); DJ1C-120W magnetic stirrer (Jiangsu Maipulong Instrument Manufacturing Co., Ltd., Changzhou, China); HH-S thermostat water bath (Jiangsu Jintan Instrument Technology Co., Ltd., Jintan, China); 101–2 series constant temperature (Shanghai Nanyang Electrical Equipment Co., Ltd., Shanghai, China); JF2204C analytical balance (Shanghai Bangxi Instrument Technology Co., Ltd. Shanghai, China); 1260 High-performance liquid chromatograph (HPLC, Agilent Technologies Co. Ltd. Santa Clara, United States); Agilent 5 HC-C8 (4.6 × 250 mm, 5 um; Agilent Technologies Co. Ltd. Santa Clara, United States) were used in the experiments.

Dexamethasone acetate powder (Tianjin Tianyao Pharmaceuticals Co., Ltd., batch number: NDAC180602); vitamin C soluble powder (Northeast Pharmaceutical Group Co., Ltd., batch number: 2617032024); cod liver oil (Sinopharm Group Co., Ltd., batch number: 20780802); vitamin B2 soluble powder (Shandong Jurong Bio Co., Ltd., batch number: 1704223); metronidazole powder (Wuhan Wuyao Pharmaceutical Co., Ltd., batch number: C04-180409); dyclonine hydrochloride powder (Hubei Xingyin Chemical Pharmaceutical Co., Ltd., batch number: 20181579); polysorbate 80 (Hunan Erkang Pharmaceutical Co., Ltd., batch number: 1720180503); polyvinyl alcohol medium viscosity (PVA medium) (Jiangxi Alpha Hi-Tech Pharmaceutical Co., Ltd., batch number: 20180701); polyvinyl alcohol low viscosity (PVA low) (Jiangxi Alpha Hi-Tech Pharmaceutical Co., Ltd., batch number: 20171102); glycerin (Guangdong Hengjian Pharmaceutical Co., Ltd., batch number 180102); ethanol (Sichuan Jinshan Pharmaceutical Co., Ltd., batch number: 20171005) were used in the experiments as reagents. All water used in the experiment was self-purified water.

### 2.2 Preparation and evaluation of the blank film

Synthetic polymer has varied adhesiveness, film formation, and water solubility characteristics with different materials (Gobi et al., 2021). Synthetic polymer film-forming materials were consequently used to prepare blank films in the present study. PVA medium (A, mg), PVA low (B, mg), and glycerol (C, mg) were selected as the main factors, and three levels were selected for each factor. The optimal composition ratios of the blank film were obtained using an orthogonal experiment, and the factor levels are shown in Supplemental Table S1. As a common film forming material, PVA has strong film forming properties. The film agent made of PVA is clean and has a short melting time, which has a great influence on the physical properties after film formation. “PVA low” means low viscosity PVA, with a viscosity of 20.0–30.0 mPa s, specifically PVA05–88, and low polymerization degree, high water solubility, good film formation, and poor ductility. “PVA medium” means PVA with medium viscosity, with a viscosity of 54.0–6.0 mPa s, specifically PVA17–88, and high polymerization degree, low water solubility, and relatively high tensile strength. The choice of two types of PVA with different viscosity or water solubility is to better and more accurately adjust PVA water solubility, film formation, and ductility.

1) An L9 (3^4^) orthogonal test was used for blank film preparation. The prescribed quantities of A and B were weighed and then swollen fully using purified water. They were then stirred at 95°C in a water bath until dissolved completely into colorless liquids. The bubbles were removed by sonication for 5 min, and A and B were then mixed at 20°C. The prescribed quantities of C were weighed, and a mixture of A and B was added, and they were then stirred and mixed. The bubbles were removed by ultrasound for 5 min, and the mixture was stored at around 25°C. Subsequently, 1 mL of blank film solution was dropped onto the coating glass board of the automatic coater. At 70 mm/s, a 0.2-μm wire rod was coated and dried naturally. The appearance evaluation indexes were softness, smoothness, film forming, and uniformity; each index was 15 points (Juliano et al., 2008). Softness: Observe the difficulty of demoulding and whether there is damage during the film uncovering process, the easier it is to demoulding, and the higher the number of no damage during the film uncovering process. Smoothness: Observe whether there are small folds and bubbles, the fewer folds and bubbles, the higher the score. Film formation: Observe the difficulty of coating film, the easier it is, the higher it is. Uniformity: Observe whether the thickness of the film is consistent, the more consistent the score is higher. The time required for the coating agent to form a film was determined. A weighted comprehensive evaluation was performed, and the scores for appearance and film-forming time accounted for 60% and 40% of the comprehensive scores, respectively (Wulf and Lewthwaite, 2016). Thus, Total score = appearance trait score (softness + smoothness + molding + uniformity) + (shortest film forming time/film forming time) ×40. The dosage ratio of each main matrix in the blank film was considered, and the film-forming material with the best proportion was called PVAa.

### 2.3 Preparation and evaluation of a drug-carrying film

According to the results of the preliminary experiment, PVAa (D, mL), dexamethasone acetate (E, mg), and metronidazole (F, mg) were selected as influence factors, and three levels of each factor were selected to obtain the optimal composition ratio of the drug-carrying film using an L9 (3^4^) orthogonal experiment; factor levels are shown in Supplemental Table S2. The prescribed quantities of E, F, vitamin C (50 mg), vitamin B2 (5 mg), tacronin hydrochloride (10 mg), and polyssorbide 80 (15 mg) were weighed. Subsequently, 5 mL of 50% ethanol was added, and the mixture was stirred. The prescribed quantity of D and cod liver oil (0.1 mL) was added, and the mixture was stirred in a water bath at 60°C until dissolved completely. It was then stored at around 25°C. Subsequently, 1 mL of the drug-carrying film solution was dropped on the coating glass plate of the automatic coater. At 70 mm/s, a 200-μm wire rod was coated and dried naturally. The evaluation methods for the drug-carrying film were the same as for the blank film.

### 2.4 Methodological determination of compound cod liver oil film former agent

The measurement conditions were as follows: the column was an Agilent 5 HC-C8 (4.6 × 250 mm, 5 um), the column temperature was 25°C, the wavelength was 275 nm, and the flow rate was 1.0 mL/min, with an injection volume of 100 μL. Sodium dihydrogen phosphate dihydrate solution-acetonitrile (65:35) (7 g/L) was used as the mobile phase.

The test solution was prepared as follows: five bottles were filled with 100 mL/bottle of the above compound cod liver oil film former solution, and 30 mL of acetonitrile was added to dissolve the sample. Afterward, it was quantitatively transferred to a 100 mL volumetric flask, diluted to the mark with acetonitrile and shaken and filtered. Subsequently, 1 mL of the filtrate was placed in a 25 mL volumetric flask, acetonitrile was added to dilute it to the mark, and it was shaken to generate the test solution. The stock solution of dyclonine hydrochloride reference substance was prepared as follows: 10 mg of dyclonine hydrochloride reference substance was placed into a 10 mL volumetric flask, dissolved and diluted to the mark with acetonitrile, and shaken. Preparation of the mixed reference substance solution was as follows: 15 mg of the dexamethasone and metronidazole reference substances were measured, respectively, into a 100 mL volumetric flask, and then 5 mL of dyclonine hydrochloride reference substance solution was added with acetonitrile to dissolve and dilute to the mark, and then shaken.

Linearity determination was as follows: 0.5, 0.8, 1.0, 1.2, and 1.5 mL of the mixed reference substance stock solution were measured into 10 mL volumetric flasks, diluted to the mark with acetonitrile, and then used as the concentration of the solution equivalent to the test solution. Subsequently, 100 μL of the above linear solution was measured for detection using the previously described chromatographic conditions. The concentration (C) and the peak area (A) were used as the abscissa and ordinate, respectively, to calculate the regression equation and the correlation coefficient.

To verify injection reproducibility, 100 μL of the solution with a concentration of 100% of the test solution was injected six times into a liquid chromatograph and then detected according to the above described chromatographic conditions. The chromatogram was recorded, and precision was determined according to the peak area.

To calculate the recovery rate, 80%, 100%, and 120% of the labeled amount of dyclonine hydrochloride, dexamethasone, and metronidazole were recovered. The 80% recovery solution contained dyclonine hydrochloride (4 mg), dexamethasone acetate reference substance (12 mg), and metronidazole reference substance (12 mg), respectively. The 100% recovery solution contained dyclonine hydrochloride (5 mg), dexamethasone acetate reference substance (15 mg), and metronidazole reference substance (15 mg). In comparison, the 120% recovery solution contained dyclonine hydrochloride (6 mg), dexamethasone acetate reference substance (18 mg), and metronidazole reference substance (18 mg), respectively. Subsequently, 100 μL of the above solution was injected into the liquid chromatograph and detected under the above described chromatographic conditions. The external standard method was used to calculate the recovery as the peak area.

For repeatability determination, 100 μL of the test and mixed reference solutions were injected six times continuously into the liquid chromatograph and detected according to the above described chromatographic conditions. The peak area ratio was recorded.

### 2.5 Interference test, separation degree and theoretical plate number test

Interference test: According to the prescription ratio, 5 g of the blank excipients were weighed accurately and placed in 100-mL measuring bottles, 60 mL acetonitrile added, ultrasonicated for 10 min, and then acetonitrile added to dilute to the scale, shaken, and filtered. Subsequently, 1 mL of the filtrate was placed into a 25 mL measuring bottle, acetonitrile added to dilute to the scale, shaken well, and the test solution obtained. According to chromatographic conditions in Section 2.4, blank excipients and blank solvents did not interfere with the content determination of this product, and the results are shown in Supplemental Figure S1.

Separation degree and theoretical plate number test: The test solution (100 μL) was assessed according to the chromatographic conditions in Section 2.4. The results are shown in Supplemental Figures S2, S3. The results indicated the theoretical plate numbers according to the dyclonine hydrochloride peak, dexamethasone peak, and metronidazole peak are 12365, 22354, and 15325, respectively. The separation degrees of dyclonine hydrochloride, dexamethasone, and metronidazole meet the requirements of content determination.

### 2.6 Statistical analysis

Our current study adopted the orthogonal experimental method to determine the optimal ratio of blank film and drug loaded film, and observed whether each factor had a significant impact on film formation. Thus, one-way Analysis (F-test) of Variance was used to test differences in continuous variables (Zhou et al., 2021; Zhu et al., 2022). In the study of the optimal proportion of blank film, the continuous variables were PVA medium, PVA low, glycerin. In the study on the optimal proportion of drug-carrying film, the continuous variables were PVAa, dexamethasone acetate, metronidazole. *p* < 0.05 was considered to indicate statistically significant difference. All statistical analyses were performed using IBM SPSS Statistics 20 (IBM Corp., Armonk, NY, United States).

## 3 Results

### 3.1 Results of the blank film orthogonal test

The influences of PVA medium (A, mg), PVA low (B, mg), and glycerol (C, mg) on the performance of the blank film matrix were as follows: A > B > C; factors A and B had a significant influence on the experimental results (*p* < .05), whereas factor C had no significant influence (*p* > .10; Tables 1, 2). The optimal composition ratio of the blank film was found to be A_3_B_2_C_2_. The blank film prepared with PVA low (55 mg), PVA medium (45 mg), and glycerol (10 mg) had the best performance.

**TABLE 1:** Results of blank film orthogonal test.

Experiment number	Factors				Appearance score	Film forming time(min)	Score of film forming time	Total score
	A(PVA medium, mg)	B(PVA low, mg)	C(glycerin, mg)	Error				
1	Level 1(35)	Level 1(35)	Level 1(6)	Level 1	24	106.9	34.79	58.79
2	Level 1(35)	Level 2(45)	Level 2(10)	Level2	40	100.1	37.17	77.17
3	Level 1(35)	Level 3(55)	Level 3(14)	Level3	26	106.9	34.79	60.79
4	Level 2(45)	Level 1(35)	Level 2(10)	Level3	36	127.4	29.21	65.21
5	Level 2(45)	Level 2(45)	Level 3(14)	Level 1	40	104.3	35.67	75.67
6	Level 2(45)	Level 3(55)	Level 1(6)	Level2	28	93.0	40.00	68.00
7	Level 3(55)	Level 1(35)	Level 3(14)	Level2	42	111.5	33.37	75.37
8	Level 3(55)	Level 2(45)	Level 1(6)	Level3	57	103.1	36.08	93.08
9	Level 3(55)	Level 3(55)	Level 2(10)	Level 1	46	106.8	34.84	80.84
Mean Level 1	65.58	66.46	73.29	71.77				
Mean Level 2	69.63	81.97	74.41	73.51				
Mean Level 3	83.10	69.88	70.61	73.03				
Range	17.51	15.51	3.780	1.75				

Note: “Mean” represents the average total score of each factor (i.e. A, B, C) at its corresponding level. The numbers in parentheses represent the amount added. For example, Mean Level 1 indicates the average value of the Total score of all Level 1 in PVA, medium. That is, “Mean Level 1”of PVA, medium = (58.79 + 77.17+60.79)/3 = 65.58. By analogy, this factor (PVA, medium) “Mean Level 2”= (65.21 + 75.67+68.00)/3 = 69.63. For the factor of “PVA, low”, “Mean Level 1”= (58.79 + 65.21+75.37)/3 = 66.46. For the factor of “glycerin”, “Mean Level 1”= (58.79 + 68.00+93.08)/3 = 73.29.

**TABLE 2:** Results of variance analysis for blank film orthogonal test.

Factor	Sum of squares of deviation	df	F	*p*
A(PVA medium)	504.51	2	103.49	<0.05
B(PVA low)	398.79	2	81.80	<0.05
C(glycerin)	22.84	2	4.69	>0.10
Error	4.88	2		

### 3.2 Orthogonal test results for the drug-carrying film

The orthogonal test results and variance analysis of the drug-carrying film are shown in Tables 3, 4. According to the results, the influences on the performance of the drug-carrying film matrix were as follows: D > E > F; factors D, E, and F had a significant influence on the experimental results (*p* < .05). The optimal composition ratio of the drug-carrying film is D_3_E_2_F_3_; that is, the drug-carrying film prepared using PVAa (3 mL), dexamethasone acetate (30 mg), and metronidazole (30 mg), had the best performance.

**TABLE 3:** Results of drug-carrying film othogonal test.

Experiment number	Factors				Appearance score	Film forming time (min)	Score of film forming time	Total score
	D (PVAa, ml)	E (dexamethasone acetate, mg)	F (metronidazole, mg)	Error				
1	Level 1(1)	Level 1(20)	Level 1(10)	Level 1	18	189.3	22.19	40.19
2	Level 1(1)	Level 2(30)	Level 2(20)	Level 2	24	133.2	31.52	55.52
3	Level 1(1)	Level 3(40)	Level 3(30)	Level 3	31	142.7	29.43	60.43
4	Level 2(2)	Level 1(20)	Level 2(20)	Level 3	27	120.5	34.86	61.86
5	Level 2(2)	Level 2(30)	Level 3(30)	Level 1	50	125.7	33.42	83.42
6	Level 2(2)	Level 3(40)	Level 1(10)	Level 2	24	105.0	40.00	64.00
7	Level 3(3)	Level 1(20)	Level 3(30)	Level 2	42	109.3	38.42	80.42
8	Level 3(3)	Level 2(30)	Level 1(10)	Level 3	48	118.1	35.57	83.57
9	Level 3(3)	Level 3(40)	Level 2(20)	Level 1	38	109.8	38.24	76.24
Mean Level 1	52.05	60.82	62.59	66.62				
Mean Level 2	69.76	74.17	64.50	66.65				
Mean Level 3	80.08	66.89	74.76	68.62				
Range	28.03	13.36	12.17	2.00				

Note: “Mean” represents the average total score of each factor (i.e. A, B, C) at its corresponding level. The numbers in parentheses represent the amount added. For example, 52.05 of “Mean Level 1” is the average “Total score” of all “Level 1” factors of “PVAa”, i.e. (40.19 + 55.52+60.43)/3 = 52.05. 60.82 was the average “Total score” of all “Level 1” of the factor “dexamethasone”, i.e. (40.19 + 61.86+80.42)/3 = 60.82. 62.59 is the average of the “Total score” of all “Level 1”of the factor “metronidazole”, i.e. (40.19 + 64.00+83.57)/3 = 62.58. And 66.62 is the average of the Total score of all Levels 1 of Error, i.e. (40.19 + 83.42+76.24)/3 = 66.62.

**TABLE 4:** Results of variance analysis for drug-carrying film orthogonal test.

Factor	Sum of squares of deviation	df	F	*p*
D(PVAa)	1205.88	2	152.49	<0.05
E(dexamethasone acetate)	267.94	2	33.88	<0.05
F(metronidazole)	256.31	2	32.41	<0.05
Error	7.91	2		

### 3.3 Validation test

Three batches of samples were prepared according to the optimal ratio of D_3_E_2_F_3_. The results are shown in Table 5. Compared with No. 5 and No. 8, the verified sample score increased with higher scores in the orthogonal test results of the drug-carrying film, and the optimized preparation process was repeatable (Table 5). The verified sample has a complete and smooth appearance, uniform thickness, uniform color, and no evident bubbles, which meets the requirements for a film agent defined in Chinese Pharmacopoeia, 2020 edition.

**TABLE 5:** Results of film formation time and appearance verification of drug-loaded film samples.

Group	No.	Softness	Smoothness	Film-forming	Uniformity	Appearance score	Film forming time (min)	Film formation time score	Total score
**Verification group**	1	14	15	13	11	53	91	36.48	89.48
	2	12	13	12	14	51	83	40.00	91.00
	3	13	14	15	14	56	88	37.73	93.73
**Drug-loaded film group**	5	10	15	12	13	50	125.7	33.42	83.42
	8	12	9	13	14	48	118.1	35.57	83.57

The drug-carrying film samples were divided into small squares of 2 × 2 cm and verified by referring to the requirements for weight difference of the film agents defined in the Chinese Pharmacopoeia, 2020 edition. According to the results in Supplemental Table S3, only one tablet exceeded the weight difference limit (±15%), and the weight difference limit was not twice the limit. The weight difference limit of the drug-loading film samples conformed with the provisions. According to the method for determining the dissolution time limit for the clotrimazole oral film in the Chinese Pharmacopoeia, 2020 edition (Queiroz et al., 2018), the drug-carrying film with the optimal ratio was cut into six films that were each 1 cm^2^ and then clamped with two layers of stainless steel wire with an inner diameter of 2.0 mm. All films should be dissolved and go through the screen within 15 min, according to the method for the determining the disintegration time limit of tablets (general rule 0921). The time spent was recorded, and the average value and RSD were calculated, as shown in Supplemental Table S4.

### 3.4 Content determination of compound cod liver oil film former

The linearity determination showed that dyclonine hydrochloride, dexamethasone acetate, and metronidazole had a good linear relationship (R ≥ 0.9999) in the concentration range of 2.5–7.5 μg/mL, 7.5–22.5 μg/mL, and 7.5–22.5 μg/mL, respectively (details not shown). The verification of injection reproducibility revealed that the RSD peak areas were 0.11% for dyclonine hydrochloride, 0.07% for dexamethasone acetate, and 0.08% for metronidazole, and they all indicated good precision (Supplemental Table S5). The average recovery rate and RSD were 100.7% and 0.24% for dyclonine hydrochloride, 99.0% and 0.70% for dexamethasone acetate, and 99.6% and 0.80% for dexamethasone acetate, respectively (Supplemental Table S6). The results of repeatability determination suggested that the repeatability RSD for dyclonine hydrochloride, dexamethasone acetate, and metronidazole were 0.83%, 0.82%, and 0.88%, respectively. All met the requirements of content determination (Supplemental Table S7).

## 4 Discussion

In the present study, dexamethasone acetate, metronidazole, vitamin C, vitamin B2, dyclonine hydrochloride, and cod liver oil were selected as the main components for the treatment of RAS. PVA was selected as the film-forming material to prepare a compound film, and its ratio was optimized using an orthogonal test. As this study has mainly discussed the optimal ratio of the preparation process, the appearance of the film agent and the film-forming time were selected as the indicators. Overall, the compound cod-liver oil film resulted in the following improvements in comparison with previous formula: (1) added vitamin C and vitamin B2; (2) replaced tetracycline with metronidazole; and (3) replaced tetracaine with dyclonine hydrochloride mucilage.

Vitamin C reduces capillary permeability and fragility, and vitamin B2 is an indispensable nutrient that maintains the integrity of the oral mucosa (Moynihan, 2008; Morelli et al., 2020). In addition, anaerobic bacteria are common in oral mucosal infections, and metronidazole has broad-spectrum anti-anaerobic bacterial activities (Reynolds-Campbell et al., 2017), which makes it an ideal anti-anaerobic bacterial agent. Moreover, dyclonine hydrochloride, a new type of safe soluble mucosal surface anesthetic, contains glycerin, which has high viscosity and adheres to the oral mucosa to enhance the anesthesia effect (Kimyon et al., 2019). The toxicity and side effects of dyclonine hydrochloride were found to be low, and the anesthesia effect was strong; the effect is thus rapid and lasting, and it can reduce allergic reactions (Kimyon et al., 2019). Compared with the compound cod-liver oil liniment, the oral immediate-release film was generally composed of the main drugs, film-forming materials, plasticizers, and flavoring agents. As a common film-forming material, PVA has strong film-forming properties. The resulting film is smooth and has a short melting time (Londhe and Umalkar, 2012), with the highest impact on the physical properties after film formation. As a common plasticizer, glycerol can increase film softness, flexibility, and humidity (Jouki et al., 2013).

The experimental results indicate that PVA low and PVA medium had significant effects on the total score for the blank film (*p* < .05). The concentration of PVA low was positively correlated with the total score, indicating that PVA low had positive effects on physical properties and film-forming time of the film agent. Therefore, it can be considered the main component of the film-forming material. The total score of the blank film after film formation first increased and then decreased with an increase in PVA medium. The reason may be that with an increase in viscosity, the flexibility and uniformity of the film were affected, and the film formation time was prolonged. The changes in glycerol had no significant effects on the total score of the blank film (*p* > .05), indicating that the glycerol mainly acts as a humectant and plasticizer and has minimal effect on its physical properties. Glycerol has a positive influence on the film-forming time, and excess glycerol can keep the surface of the film agent wet and prolong the film-formation time (Jouki et al., 2013).

Compared with traditional research methods that only consider the film-forming material, the current study also investigated the effects of the main drugs on the physical properties of the film. The results suggest that the main drugs (i.e., PVAa, dexamethasone acetate, and metronidazole) had specific impacts on the physical properties of the film. As the different main drugs have different binding degrees and binding methods with the film-forming material, they have different effects on the physical properties and film-forming time of the film agent. Although the main drugs had an impact on the physical properties of the film, they were not ideal as the film-forming material, and this combination may have an impact on the dissolution rate of the main drugs. According to the orthogonal test results, with an increase in dexamethasone acetate, the total score first increased and then decreased after film formation. The results may also be because the solubility of dexamethasone acetate reached saturation, and the insoluble particles increased, which affected the film formation and appearance score (Chen et al., 2008; Silva et al., 2018). The PVAa was positively correlated with the total score of the drug-loaded film after film formation, suggesting that the effect of the film-forming agent on its total score can be observed as a whole rather than individually after the optimal ratio of film-forming materials is obtained.

Overall, the results indicate that the compound cod liver oil film prepared in the present study has an optimal appearance and meets the relevant requirements defined in the Chinese Pharmacopoeia, 2020 edition. This also provides a solid foundation for future research to investigate the associated quality control methods and pharmacokinetic parameters.

The present study had some limitations. First, the experimental conditions cannot fully simulate the *in vivo* environment, so that the effects of the compound cod liver oil film former agent on the human body require further verification. Second, only the scores for appearance and film-forming time were calculated for a few sets of doses of blank film and main drugs and the specific doses were not sufficiently refined. Third, whether changes in the external physical environment had a specific effect on the results was not examined.

## 5 Conclusion

The present study has proposed an optimized compound cod liver oil film former agent and an appropriate preparation method. An orthogonal experiment was adopted and the appearance and film-forming time of the film coating agents were used as the indicators. The ratios for the preparation process were optimized. According to the results, compound cod liver oil film former agent has good performance, which highlights the value of this research method. The clinical effects of the new agent in patients with RAS requires further investigation.

## Data Availability

The raw data supporting the conclusion of this article will be made available by the authors, without undue reservation.
