# Supplementary material for: Optimization and preparation of a compound cod liver oil film former agent: an orthogonal design
Source: Front Pharmacol. 2024 Mar 1;15:1296448. doi: 10.3389/fphar.2024.1296448 (PMC10940503; doi:10.3389/fphar.2024.1296448)
Supplement: Supplementary file 1 [file DataSheet1.docx]

**Supplemental Table 1. Levels of blank film factor**

| Level | Factors | | |
| --- | --- | --- | --- |
|  | A (PVA medium, mg) | B (PVA low, mg) | C (glycerin, mg) |
| 1 | 35 | 35 | 6 |
| 2 | 45 | 45 | 10 |
| 3 | 55 | 55 | 14 |

**Note:** “A” means PVA medium (mg), “B” means PVA low (mg), and “C” means glycerol (mg). "1, 2, and 3"are the three levels of the above three factors, respectively. Appearance evaluation indexes are softness, smoothness, film formation and uniformity, with 15 points for each index. Softness: Observe the difficulty of demoulding and whether there is damage during the film uncovering process, the easier it is to demoulding, and the higher the number of no damage during the film uncovering process. Smoothness: Observe whether there are small folds and bubbles, the fewer folds and bubbles, the higher the score. Film formation: Observe the difficulty of coating film, the easier it is, the higher it is. Uniformity: Observe whether the thickness of the film is consistent, the more consistent the score is higher. The time required for the coating agent to form a film was determined. A weighted comprehensive evaluation was performed, and the scores for appearance and film-forming time accounted for 60% and 40% of the comprehensive scores, respectively.

**Supplemental Table 2. Levels of drug-carrying film factors**

| Level | Factor | | |
| --- | --- | --- | --- |
|  | D (PVAa, ml) | E (dexamethasone acetate, mg) | F (metronidazole, mg) |
| 1 | 1 | 20 | 10 |
| 2 | 2 | 30 | 20 |
| 3 | 3 | 40 | 30 |

**Supplemental Table 3. Verification results of weight difference of drug-carrying film samples**

| **No.** | **Weight (mg)** | **difference from mean (mg)** | **The difference from the mean as a percentage of the mean** |
| --- | --- | --- | --- |
| 1 | 2.6 | 0.15 | 5.91% |
| 2 | 2.4 | -0.06 | -2.24% |
| 3 | 2.5 | 0.05 | 1.83% |
| 4 | 2.7 | 0.25 | 9.98% |
| 5 | 2.1 | -0.36 | -14.46% |
| 6 | 2.1 | -0.36 | -14.46% |
| 7 | 2.6 | 0.15 | 5.91% |
| 8 | 2.4 | -0.06 | -2.24% |
| 9 | 2.3 | -0.16 | -6.31% |
| 10 | 2.5 | 0.05 | 1.83% |
| 11 | 2.8 | 0.35 | 14.05% |
| 12 | 2.0 | -0.46 | -18.53% |
| 13 | 2.1 | -0.36 | -14.46% |
| 14 | 2.4 | -0.06 | -2.24% |
| 15 | 2.7 | 0.25 | 9.98% |
| 16 | 2.7 | 0.25 | 9.98% |
| 17 | 2.7 | 0.25 | 9.98% |
| 18 | 2.4 | -0.06 | -2.24% |
| 19 | 2.5 | 0.05 | 1.83% |
| 20 | 2.6 | 0.15 | 5.91% |
| Mean | 2.455 |  |  |

**Supplemental Table 4. Validation results of the dissolution time of the drug-carrying film samples**

| **Dissolution time limit** | | | | | | | **Mean** | **RSD** |
| --- | --- | --- | --- | --- | --- | --- | --- | --- |
| No. | 1 | 2 | 3 | 4 | 5 | 6 |  |  |
| Verification group | 14.76 | 10.61 | 12.37 | 13.43 | 11.57 | 14.07 | 12.80 | 1.57 |

**Supplemental Table 5 Results of precision determination**

| **No.** | **Dyclonine** | **Dexamethasone acetate** | **Metronidazole** |
| --- | --- | --- | --- |
| 1# | 407.3 | 581.1 | 602.6 |
| 2# | 408.2 | 582.1 | 603.4 |
| 3# | 407.1 | 581.1 | 602.3 |
| 4# | 407.0 | 581.1 | 602.1 |
| 5# | 407.1 | 581.1 | 602.3 |
| 6# | 407.1 | 581.2 | 605.5 |
| Means | 407.3 | 581.3 | 602.5 |
| RSD% | 0.11 | 0.07 | 0.08 |

**Supplemental Table 6. Results of recovery rate determination**

| **No.** | | **Added amount（mg）** | **Measured amount（mg）** | **Recovery rate（%）** | **Average Recovery rate（%）** | **RSD%** |
| --- | --- | --- | --- | --- | --- | --- |
| **Dyclonine** | | | | | | |
| 80% | 1# | 3.97 | 4.00 | 100.8 | 100.7 | 0.24 |
|  | 2# | 3.97 | 4.00 | 100.7 |  |  |
|  | 3# | 3.97 | 3.98 | 100.3 |  |  |
| 100% | 1# | 4.96 | 5.01 | 100.9 |  |  |
|  | 2# | 4.96 | 4.98 | 100.4 |  |  |
|  | 3# | 4.96 | 5.00 | 100.8 |  |  |
| 120% | 1# | 5.96 | 6.00 | 100.8 |  |  |
|  | 2# | 5.96 | 5.99 | 100.6 |  |  |
|  | 3# | 5.96 | 6.02 | 101.6 |  |  |
| **Dexamethasone acetate** | | | | | | |
| 80% | 1# | 12.18 | 12.09 | 99.3 | 99.0 | 0.70 |
|  | 2# | 12.33 | 12.14 | 98.4 |  |  |
|  | 3# | 12.19 | 12.05 | 98.9 |  |  |
| 100% | 1# | 15.04 | 15.11 | 100.5 |  |  |
|  | 2# | 15.43 | 15.16 | 98.2 |  |  |
|  | 3# | 15.66 | 15.46 | 98.7 |  |  |
| 120% | 1# | 18.13 | 18.05 | 99.6 |  |  |
|  | 2# | 18.21 | 17.98 | 98.7 |  |  |
|  | 3# | 18.23 | 17.97 | 98.6 |  |  |
| **Metronidazole** | | | | | | |
| 80% | 1# | 12.89 | 12.68 | 98.4 | 99.6 | 0.80 |
|  | 2# | 12.65 | 12.65 | 100.0 |  |  |
|  | 3# | 12.12 | 12.06 | 99.5 |  |  |
| 100% | 1# | 15.10 | 15.03 | 99.5 |  |  |
|  | 2# | 15.00 | 14.93 | 99.5 |  |  |
|  | 3# | 14.99 | 14.79 | 98.7 |  |  |
| 120% | 1# | 18.66 | 18.77 | 100.6 |  |  |
|  | 2# | 18.20 | 18.15 | 99.7 |  |  |
|  | 3# | 18.11 | 18.27 | 100.9 |  |  |

**Supplemental Table 7. Results of** **repeatability determination**

| **No.** | **Content（%）** | **Average content（%）** | **RSD%** |
| --- | --- | --- | --- |
| **Dyclonine** | | | |
| 1# | 99.6 | 99.7 | 0.83 |
| 2# | 100.1 |  |  |
| 3# | 99.1 |  |  |
| 4# | 101.1 |  |  |
| 5# | 99.9 |  |  |
| 6# | 98.7 |  |  |
| **Dexamethasone acetate** | | | |
| 1# | 100.5 | 100.2 | 0.82 |
| 2# | 98.7 |  |  |
| 3# | 100.7 |  |  |
| 4# | 100.8 |  |  |
| 5# | 99.8 |  |  |
| 6# | 100.7 |  |  |
| **Metronidazole** | | | |
| 1# | 99.3 | 99.7 | 0.88 |
| 2# | 99.1 |  |  |
| 3# | 98.6 |  |  |
| 4# | 99.6 |  |  |
| 5# | 100.2 |  |  |
| 6# | 101.1 |  |  |


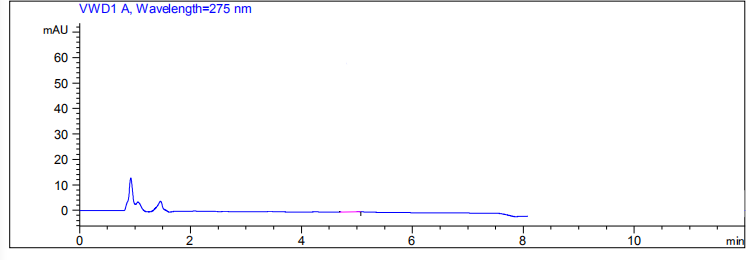


**Supplemental Figure 1. The results of blank test**

**
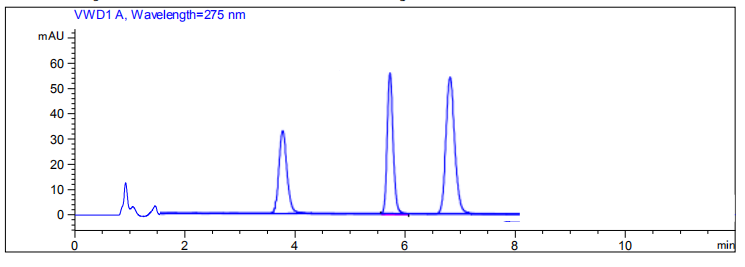
**

1

2

3

**Supplemental Figure 2. Separation degree of test products**

**1. Dacronine hydrochloride; 2. Dexamethasone; 3. Metronidazole**


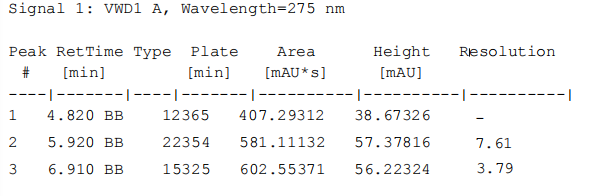


**Supplemental Figure 3. Theoretical plate number of test products**
